# RB inactivation in keratin 18 positive thymic epithelial cells promotes non-cell autonomous T cell hyperproliferation in genetically engineered mice

**DOI:** 10.1371/journal.pone.0171510

**Published:** 2017-02-03

**Authors:** Yurong Song, Teresa Sullivan, Kimberly Klarmann, Debra Gilbert, T. Norene O’Sullivan, Lucy Lu, Sophie Wang, Diana C. Haines, Terry Van Dyke, Jonathan R. Keller

**Affiliations:** 1 Mouse Cancer Genetics Program, Center for Cancer Research, National Cancer Institute, Frederick, Maryland, United States of America; 2 Basic Science Program, Leidos Biomedical Research, Inc., Frederick National Laboratory for Cancer Research, Frederick, Maryland, United States of America; 3 Pathology/ Histotechnology Laboratory, Leidos Biomedical Research, Inc., Frederick National Laboratory for Cancer Research, Frederick, Maryland, United States of America; Tokyo Daigaku, JAPAN

## Abstract

Thymic epithelial cells (TEC), as part of thymic stroma, provide essential growth factors/cytokines and self-antigens to support T cell development and selection. Deletion of Rb family proteins in adult thymic stroma leads to T cell hyperplasia *in vivo*. To determine whether deletion of Rb specifically in keratin (K) 18 positive TEC was sufficient for thymocyte hyperplasia, we conditionally inactivated Rb and its family members p107 and p130 in K18+ TEC in genetically engineered mice (*TgK18GT*_*121*_*; K18* mice). We found that thymocyte hyperproliferation was induced in mice with Rb inactivation in K18+ TEC, while normal T cell development was maintained; suggesting that inactivation of Rb specifically in K18+ TEC was sufficient and responsible for the phenotype. Transplantation of wild type bone marrow cells into mice with Rb inactivation in K18+ TEC resulted in donor T lymphocyte hyperplasia confirming the non-cell autonomous requirement for Rb proteins in K18+ TEC in regulating T cell proliferation. Our data suggests that thymic epithelial cells play an important role in regulating lymphoid proliferation and thymus size.

## Introduction

T cell development and maturation is regulated, in part, by thymic stroma, which provide signals for pro T cell differentiation. Thymic stroma is very heterogeneous, consisting of cortical thymic epithelial cells (cTEC), medullary thymic epithelial cells (mTEC), fibroblasts, macrophage, dendritic and endothelial cells [1, 2]. Epithelium usually can be characterized by keratin (K) expression [3–5]. Keratins are cytoskeleton protein intermediate filaments assembled from heterodimeric subunits of acidic type I and basic type II proteins. Acidic type I keratins (K9- K28) are usually coexpressed with their heterodimeric subunits of basic type II keratins (K1-K8, and K71- K80) (e.g. K18 paired with K8, and K14 with K5). Type I K18 usually is paired with type II K8 and mainly expressed in epithelial tissues. cTEC express Ly51 and K8/18 with a minor population co-expressing both K8/18 and K5, which regulate positive selection of T lymphocytes by self-antigen presentation [6–9]. mTEC are Ly51^-^ and express K5 as well as low levels of K8/18 [7–11], and regulate negative selection of T lymphocytes by tissue-restricted antigen expression in order to establish self-tolerance [12]. While it is known that thymic stroma produces cytokines and growth factors (e.g. receptor ligands and growth factors such as Notch ligands, c-KIT ligand, Hedgehog, IL-7, CCL21, and CXCL12), and signals that regulate T cell survival and proliferation, the precise contribution of thymic epithelial subtypes to T cell development is unknown [13].

Rb and its family members (p107 and p130) are central regulators of the cell cycle. It has been demonstrated previously that inactivation of Rb tumor suppression (Rb-TS) (Rb and its family members p107 and p130) in multiple epithelial tissues and brain astrocytes initiates tumorigenesis in genetically engineered mice (GEM) by increasing proliferation and apoptosis mainly through a cell-autonomous mechanism [14–17]. The role of Rb in hematopoietic system has been extensively examined by crossing conditional RB knockout mice with or without its family members p130 and p107 to Mx1-Cre transgenic mice driven by type I interferon (IFN)-α/β-inducible Mx1 promoter via intraperitoneal injection of polyinosinic-polycytidylic acid (pI-pC), a synthetic double-stranded RNA that induces expression of endogenous IFN [18]. Thus, Cre recombination occurs in cells expressing the IFN receptor, including hematopoietic cells, monocytes, microphages, and mesenchymal cells. Deletion of RB by Mx1-Cre led to myeloproliferation through the mechanism of RB-dependent interaction between myeloid-derived cells and bone marrow (BM) microenvironment, since RB loss from either hematopoietic cells, or niche cells alone was insufficient to promote myeloproliferation [19]. However, additional deletion of p130 on p107 null background led to early death at 3–6 weeks of age due to hyperproliferation of multiple organs [20–22]. Surprisingly, p107 heterozygous mice with deletion of both RB and p130 survived, but had enlarged thymuses with increased cellularity [23]. Bone marrow transplantation studies demonstrated that T cell hyperplasia resulted from non-cell-autonomous loss of Rb proteins in thymic stroma. However, it is not clear which epithelial subtype contributes to the phenotype since Mx1-Cre is expressed in multiple subtypes of thymic stroma. To determine if Rb inactivation specifically in K18+ TEC accounts for the T cell hyperplasia, we utilize a Cre-inducible transgenic mouse model, which conditionally expresses the first 121 amino acids of SV40 T antigen (T_121_) in specific K18 subtype (*TgK18GT*_*121*_; *K18* mice) [24]. Breeding *TgK18GT*_*121*_ mice with mice expressing Cre-recombinase inactivated all three Rb family members in K18-expressing TEC. We found that inactivation of Rb-TS in K18+ TEC is sufficient to promote T cell proliferation in a non-cell autonomous manner without disrupting T cell development.

## Materials and methods

### Mice

*K18* mice (*TgK18GT*_*121*_) [24] were crossed to *β-actin Cre* [25], *R26CreER* [26], or *PbCre4* [27] mice (S1 Text). Background recombination was observed in *K18;R26CreER* mice without tamoxifen treatment, which was sufficient to induce the transgene expression. Thus, *K18;R26CreER* mice were not treated with tamoxifen. *R26YFP* mice [28] were crossed to *β-actin Cre* transgenic mice to harvest T cells for FACS analysis at 2 months of age. All bone marrow transplantation recipients were pretreated with acid water and antibiotics seven days before transplantation. Animals that did not receive bone marrow cells were moribund 12–15 days after irradiation due to failure of hematopoietic reconstitution.

### Ethics statement

This study was carried out in strict accordance with the recommendations in the Guide for the Care and Use of Laboratory Animals of the National Institutes of Health. The protocol was approved by the Institutional Animal Care and Use Committees (ACUC) at the University of North Carolina-Chapel Hill and at the National Cancer Institute (NCI)-Frederick (Permit Number: 11–030). All animals in this study were monitored daily and provided wet food when mice showed early evidence of sickness. Unexpected deaths were not observed. All mice were euthanized by CO2 asphyxiation per the “Guidelines For the Euthanasia of Mouse and Rat Fetuses and Neonates” as defined by the ACUC of UNC-Chapel Hill and NCI-Frederick to minimize pain and suffering. Humane endpoints were used for all survival studies as defined by the ACUC Guidelines for Experimental Neoplasia (e.g. rapid respiration or difficulty breathing; rough coat combined with reduced activity levels; impaired eating, drinking, or defecating; rapid weight loss greater than 20% of the original baseline body weight; and presence of a visible mass or palpable mass up to 2 cm in diameter).

### Histopathology and immunostaining

Thymuses were dissected and fixed overnight in 10% neutral buffered formalin, transferred to 70% ethanol, and routinely processed and embedded in paraffin. Four μm sections were stained with haematoxylin and eosin (H.E.) for histopathological examination. Immunohistochemistry (IHC) and immunofluorescence (IF) analyses were performed as previously described [14]. Antibodies included: anti-K8/18 (1:500, Guinea Pig polyclonal, GP11, Progen Biotechnik, GMBH, Heidelberg, Germany), anti-K19 (1:500, Rabbit monoclonal, Epitomics, CA), anti-K5 (1:3000, Rabbit polyclonal, PRB-160P, BioLegend, Dedham, MA), anti-GFP (1:200, monoclonal, b-2, Santa Cruz), anti-Ki67 (1∶500, rabbit polyclonal, 06–570, BD Pharmingen, San Diego, CA), and anti-SV40 T antigen (1:100, mouse monoclonal, DP02-200UG, Calbiochem). For double or triple IF staining, the first primary antibody (anti-K8/18) was incubated for 2 hours at room temperature followed by the second and third primary antibody (anti-GFP, anti-K5, anti-Ki67, and/or anti-T_121_) incubation overnight. Mixed Alexa fluor 488, 594, and 633 (1:200 dilution, Invitrogen) served as secondary antibodies. Nuclei were stained with DAPI. Images were captured using Zeiss light, immunofluorescence, or confocal microscopes.

### Flow cytometry

T lymphocytes were mechanically dissociated from thymus using frosted glass slides in DMEM with 5% FBS. Red blood cells were lysed using ACK buffer, and passed through 40 μm mesh filter. T cells were incubated with Fc Block (BD Biosciences) in 3% fetal bovine serum (FBS) (v/v) in PBS for 20 minutes on ice. 1–2 million cells were incubated with or without fluorescent-conjugated antibodies that recognize CD4, CD8, CD45, B220, or isotype control antibodies (BD Biosciences) in the dark for 30 minutes at 4°C. Cells were washed 3x and resuspended in 1% FBS (v/v) in PBS. For FACS sorting, CD45 stained cells were sorted using BD FACSAria II SORP cell sorter (BD Biosciences). CD45+ T cells and CD45- thymic stromal cells were subjected to RNA extraction using Ambion RiboPure RNA purification Kit (Thermo Fisher Scientific) and RT-PCR (Supplementary Methods). For CD4 and/or CD8-stained cells, they were fixed with paraformaldehyde at a final concentration of 1% (v/v). Cells were then run on Dako CyAn ADP flow cytometer or BD FACSCanto II Analyzer, and analyzed using FlowJo software (FlowJo, LLC., Ashland, OR). At least 30,000 viable events were collected for analysis.

### Bone marrow transplantation

Irradiated (10 Gy) 3 month old Ly5.2+ *C57BL/6* (*WT*) recipients were transplanted with 2x10^6^ bone marrow cells from either Ly5.1+ *K18;PbCre4* (*K18*) or *WT* mice by tail vein injection. The majority of the bone marrow cells used in our transplantation studies include hematopoietic stem and progenitor cells and mature hematopoietic cells (erythroid, myeloid, and B cells). In addition, this population contains very few stromal cells of mesenchymal and endothelial lineages. For reciprocal transplantations, Ly5.2+ *WT* bone marrow cells were transplanted into irradiated Ly5.1+ *K18;PbCre4* (*K18)* or *WT* recipients.

### Statistical analyses

Student t test was performed to evaluate the statistical significance. P < 0.05 was considered statistically significant.

### Additional methods

RT-PCR and CBC analysis are described in the supplementary information (S1 Text).

## Results and discussion

### Transgene is expressed in K18 positive TEC

**Fig 1:**
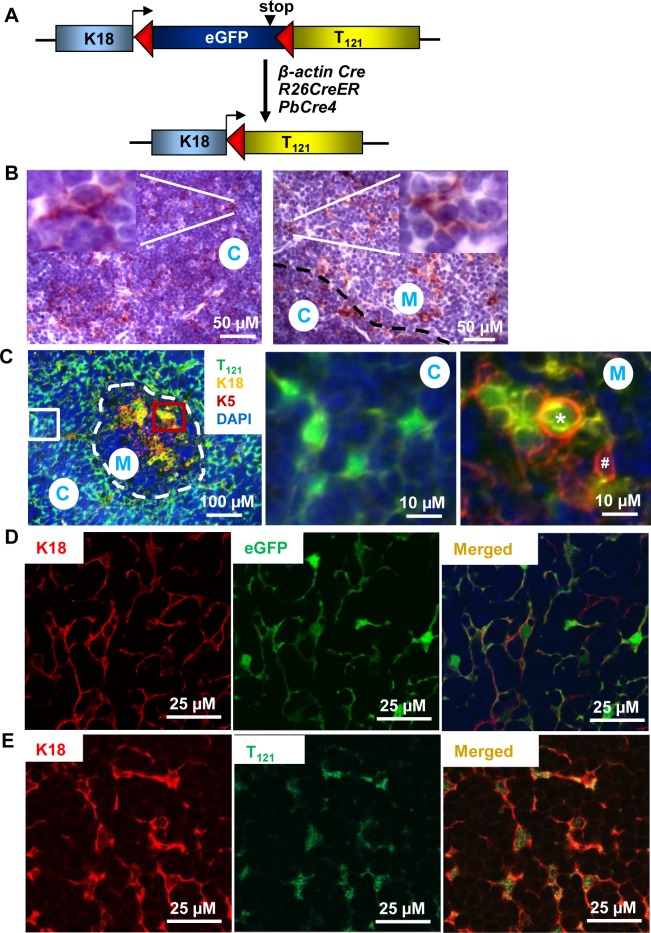
Transgene expression by immunostaining in *TgK18GT*_*121*_ (*K18*) or induced-*K18* mice. (A) Transgene cassette consisting of floxed eGFP stop cassette upstream of truncated SV40 large T antigen (first 121 amino acid; T_121_) was inserted into the 1^st^ exon of K18 gene on a bacterial artificial chromosome (BAC). Transgene eGFP was driven by K18 regulation. Once K18 mice were crossed to a transgenic mice expressing Cre recombinase, T_121_ was expressed directly under K18 regulation. (B) Representative images of K18 IHC staining in cortex (C) and medulla (M) of *WT* thymus. Inserts are higher magnification of the images. (C) Representative immunofluorescence images of T_121_ (green), K18 (yellow), K5 (red), and DAPI (blue) in cortex (C) and medulla (M) delineated with a white dotted line, in induced-*K18;Cre* thymus. Middle and right images are higher magnification of areas in white and red boxes of left image, respectively. Right image: * Cell is positive for T_121_, K18, and K5, and # positive for K5 only. (D) Representative images of K18 (red) and eGFP (green) immunostaining in thymic cortex and medulla (data not shown) of uninduced-*K18* mice (Cre negative). (E) Representative images of K18 (red) and T_121_ (green) immunostaining in thymic cortex and medulla (data not shown) of induced-*K18* mice (*K18;β-actin Cre*).

Keratins are widely used to characterize epithelial tissues including thymus [3]. First, we assessed keratin expression in thymic cortex and medulla by immunohistochemistry (IHC). K18 was highly expressed in cTEC and junction of cTEC and mTEC, and less in mTEC (Fig 1B), and K5 and K19 were expressed predominantly in mTEC (S1A Fig). This is consistent with previous reports [6, 7, 10, 11]. To determine the impact of Rb inactivation in K18+ thymic epithelial cells on T cell development, we inactivated Rb and its family members p107 and p130 (Rb tumor suppression; Rb-TS) in K18+ TEC by using a Cre-inducible K18-driven model (*TgK18GT*_*121*_; *K18* mice), in which loxP-flanked eGFP stop cassette upstream of truncated SV40 T antigen (1^st^ 121 amino acid; T_121_) was driven by K18 regulation in a bacterial artificial chromosome (Fig 1A) [24]. As predicted, eGFP was mainly expressed in cortical thymus and coexpressed with K18 in TEC of *TgK18GT*_*121*_ mice (Fig 1D). To determine the impact of Rb-TS inactivation in K18+ TEC, we crossed *TgK18GT*_*121*_ mice to mice ubiquitously expressing Cre-recombinase (*β-actin Cre* and *R26CreER)* (Fig 1A). Transgene T_121_ was expressed in cTEC and junction of cTEC and mTEC with less expression in medulla (S1B Fig). Double/triple immunostaining showed T_121_ was coexpressed with K18 in cortex and also medulla (Fig 1C and 1E). Interestingly, we observed that few T_121_-expressing cells were co-stained with both K18 and K5 in medulla (Fig 1C, Right Panel *), but other K5+ cells were negative for K18 and T_121_ (Fig 1C Right Panel #). Moreover, some medullary K18+ T_121_-expressing cells occasionally formed small glandular structures surrounded by K5+ cells (S2C Fig), suggesting that the K18+ T_121_-expressing cells were proliferating. Thus, T_121_ transgene was targeted to K18+ thymic epithelial cells.

### Rb-TS inactivation in K18+ TEC leads to thymic hyperproliferation

**Fig 2:**
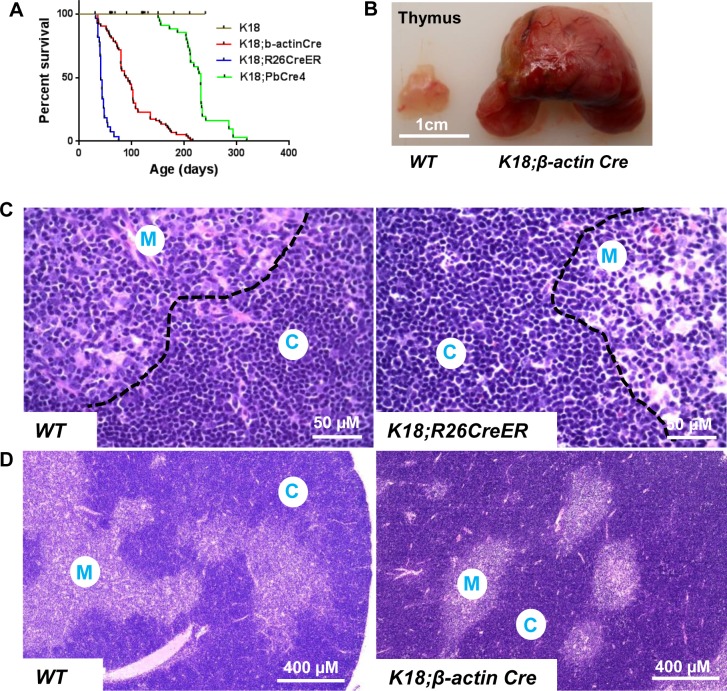
Rb-TS inactivation in K18+ TEC led to decreased survival and thymic hyperplasia. (A) Kaplan-Meier survival curve of *K18;β-actin Cre* (n = 74), *K18;R26CreER* (n = 27), and *K18;PbCre4* (n = 45) mice with median survival of 94, 41, and 231 days, respectively. Uninduced -*K18* mice (n = 8) did not develop any gross abnormalities. (B) Gross phenotype of thymuses in *WT* and *K18;β-actin Cre* mice. (C) Representative images of H.E. stained thymus sections in *WT*, *K18;R26CreER* mice. C: cortex; M: medulla. (D) Representative low magnification images of H.E. stained thymuses in *WT* and *K18;β-actin Cre* mice. C: cortex; M: medulla.

Inactivation of Rb-TS in K18+ TEC in *K18;β-actin Cre* and *K18;R26CreER* mice resulted in median survival of 94 and 41 days, respectively (Fig 2A). All mice had enlarged thymuses which compressed the lungs, and was the cause of death (Fig 2B). To exclude the possibility that Rb-TS inactivation during embryogenesis caused this phenotype, we crossed *TgK18GT*_*121*_ to *PbCre4 mice* [27], where low levels of Cre-recombinase were detected in adult thymuses (2 month) by RT-PCR (S2A Fig). As predicted, T_121_ expression was induced mainly in cortex with some expression in medulla (S1B Fig, right panel). Time course study in *TgK18GT*_*121*_;*PbCre4* mice revealed that T_121_ was induced in 2 month, but not 1 month old thymuses (S1C Fig), which may be due to no to very low expression of Cre in thymuses of 1 month old *TgK18GT*_*121*_;*PbCre4* mice. Expression of T_121_ led to enlarged thymuses but longer median survival (231 days) (Fig 2A), indicating that this phenotype was not due to embryonic inactivation of Rb-TS.

**Fig 3:**
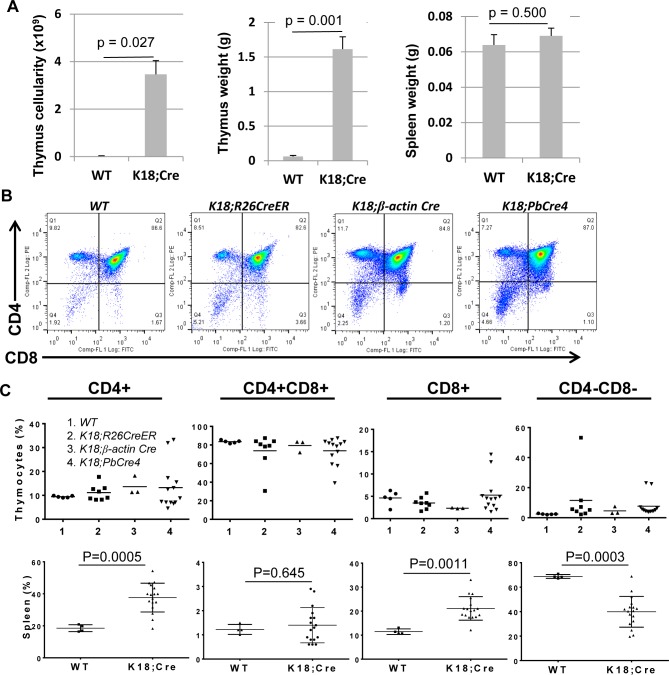
T cell populations are not altered by Rb-TS inactivation in K18+ TEC. (A) Total thymic cellularity (left); thymus weight (middle) and spleen weight (right) in grams (g) in *WT* (n = 5) and *K18;Cre* (n = 10) mice. Data are presented as mean ± SEM. p<0.05 is considered statistically significant. (B) Representative FACS plots of CD4 and CD8 staining in *WT*, *K18;R26CreER*, *K18;β-actin Cre*, and *K18;PbCre4* thymuses. (C) Percentage of CD4+, CD4+CD8+, CD8+, and CD4-CD8- T cell subpopulations in thymuses of *WT* (1, n = 5), *K18;R26CreER* (2, n = 8), *K18;β-actin Cre* (3, n = 3), and *K18;PbCre4* (4, n = 13) (top, no statistically significant difference among cohorts), and T cell subpopulations in spleens (bottom) of *WT* (n = 4) and *K18;Cre* (n = 17) by FACS analysis. p<0.05 is considered statistically significant.

Histopathology of induced-*K18* mice showed thymic hyperplasia (increased overall size of thymus compared to wildtype thymus) with low incidence of T-cell lymphoblastic lymphoma (2%) (S2B Fig). Overall thymic architecture (cortex vs. medulla proportions) was not altered (Fig 2C and 2D), and the thymic enlargement was correlated with increased thymus weight and cellularity (Fig 3A). Ki67 staining revealed hyperproliferation of both cortical and medullary thymic epithelial cells (S3 Fig), which was the result of T_121_ expression in K18+ TEC. In addition, lymphocytes were also proliferating (S3 Fig). This was likely due to non-cell autonomous effect of proliferating TEC. CBC analysis showed 50% increase of white blood cell count (WBC) in induced-*K18* mice compared to wildtype controls (Table 1, p = 0.0892), indicating a possibility of increased T cells output from thymus to peripheral, and/or higher survival rate of T cells in the blood of *K18;Cre* mice compared to wildtype. Other parameters in CBC panel measured had no significant difference between *WT* and *K18;Cre* mice (Table 1).

Immunophenotypic analysis of thymic T cell populations showed that the percentages of CD4+, CD8+, CD4+CD8+, and CD4-CD8- were not affected (Fig 3B and 3C top), except 2% of mice that developed lymphoma (S2D Fig). This might be the result of spontaneous genetic events (e.g. mutations or translocations) in a small number of highly proliferating immature T cells. We observed two-fold increase of splenic CD4+ and CD8+ cells in *K18;Cre* mice compared to wildtype by FACS analysis (Fig 3C bottom). This is consistent with the study by Klug et al. that the number of splenic T cells increased 1.5 fold in K5-driving cyclin D1 transgenic mice, which showed similar thymic hyperplasia as *K18;Cre* mice [29]. However, increased splenic T cells did not lead to increased spleen weight (Fig 3A right). This is highly likely due to decreased splenic B220+ cells (S4B Fig and discussion later).

**Table 1:** CBC profile in whole blood of Wildtype and K18;Cre mice.

CBC panel	Wildtype (n = 5)	K18;Cre (n = 20)
White Blood Cell count (WBC, 10e3/uL)	5.2±0.7	7.8±0.7
Lymphocyte (%)	81.0±0.9	77.8±1.8
Granulocyte (%)	11.9±0.6	12.7±1.4
Monocyte (%)	7.1±0.4	8.5±0.5
Hematocrit (HCT, %)	40.2±1.7	37.6±1.4
Mean Corpuscular Volume (MCV, fL)	48.3±1.8	43.9±0.4
Red Blood Cell count (RBC, 10e6/uL)	8.3±0.2	8.1±0.5
Hemoglobin (Hb, g/dL)	12.9±0.3	13.6±0.5
Mean Corpuscular Hemoglobin (MCH, pg)	15.5±0.1	15.9±0.3
Mean Corpuscular Hemoglobin Concentration (MCHC, g/dL)	32.3±1.1	37.5±1.5
Red cell Distribution Width (RDW, %)	15.9±1.0	18.8±0.3
Mean Platelet Volume (MPV, fL)	6.1±0.1	6.3±0.1
Platelet Count (PLT, 10e3/uL)	716.8±40.8	537.9±47.3

### Transgene is not expressed in thymocytes

**Fig 4:**
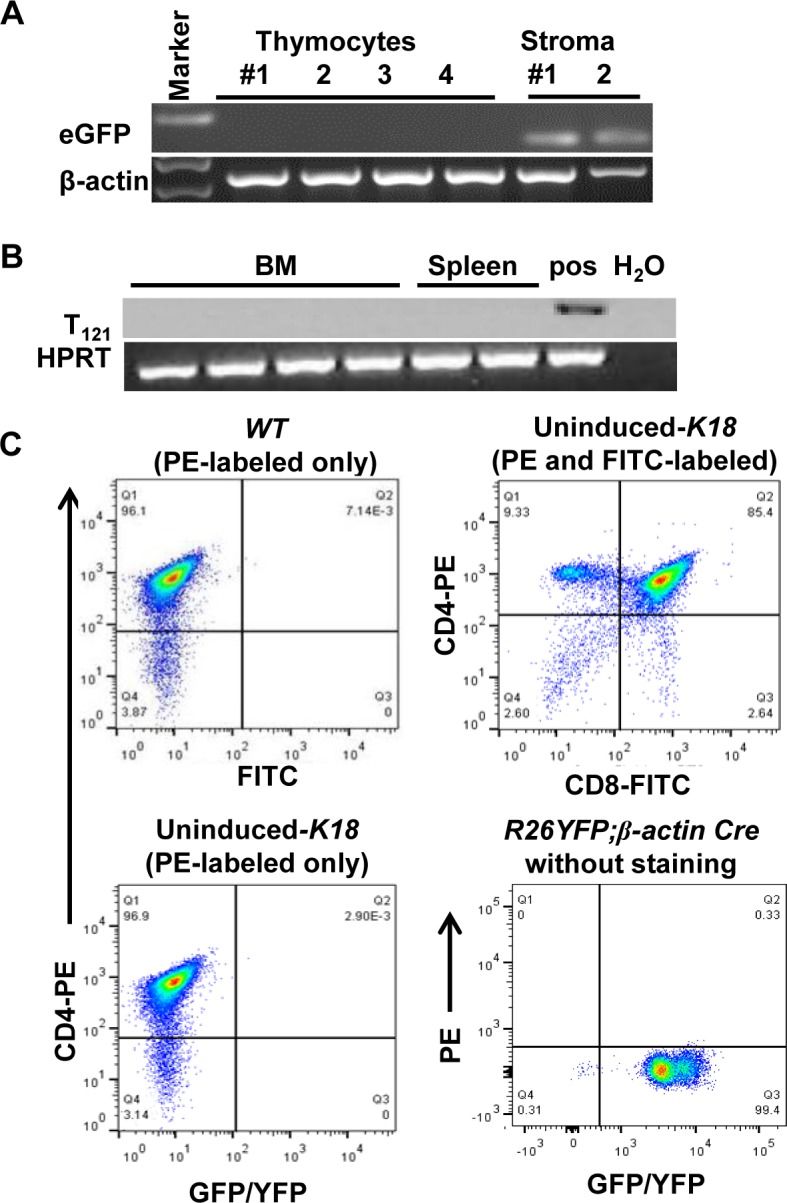
Transgene is not expressed in thymocytes. (A) eGFP mRNA levels in thymocytes and thymic stroma of uninduced-*K18* mice by RT-PCR. Thymocytes was released by gently applying pressure on thymus between two frosted glass slides, and then FACS-sorted for CD45+ cells. CD45- cells were used as thymic stroma and served as positive control. RNA was extracted from both CD45+ thymocytes and CD45- thymic stroma for RT-PCR. β-actin was used as loading control. (B) T_121_ mRNA levels in bone marrow (BM) and spleen of *K18;PbCre4* mice by RT-PCR. Bone marrow cell suspension was obtained by flushing the femurs with PBS. CD45- thymic stroma obtained for Fig 4A was used as positive control (pos), and HPRT as loading control. (C) Representative FACS plot to assess whether eGFP was expressed in thymocytes of uninduced-*K18* mice. Thymocytes were stained with only CD4-PE antibody in *WT* and uninduced-*K18* mice (left panel), or with both CD4-PE and CD8-FITC antibodies in uninduced-*K18* mice (top right). YFP in thymocytes of *R26YFP;β-actin Cre* mice without any antibody staining was readily detected and used as a positive control (bottom right).

To exclude the possibility that thymic hyperplasia phenotype observed in Cre-induced *K18* mice was due to unexpected transgene expression in T cells, we isolated CD45+ thymocytes from uninduced-*K18* thymus by FACS sorting. We could not detect green/GFP using a stereo fluorescence microscope in these thymocytes. However, we did observe green/GFP in thymic stroma (data not shown). Consistently, CD45+ thymocytes did not express eGFP mRNA by RT-PCR, while thymic stroma did (Fig 4A), demonstrating that transgene expression was specifically targeted to TEC. Furthermore, we assessed T_121_ mRNA in bone marrow (BM) or spleen in Cre-induced *K18* mice since all thymocytes were derived from BM (Fig 4B). Consistent with the eGFP mRNA not expressed in thymocytes, we did not detect T_121_ mRNA in both bone marrow and spleen, suggesting that transgene was not expressed in thymocytes. In addition, T_121_ and K18 were coexpressed in cultured TEC from Cre-induced *K18* mice (S2E Fig). Moreover, we performed flow cytometry analysis to determine if there were any eGFP+ thymocytes in uninduced-*K18* mice (Fig 4C). We examined CD4+ and CD4- cells for GFP/YFP expression, and did not detect any GFP/YFP positive CD4+ or CD4- thymocytes in the uninduced-*K18* mice (Fig 4C, bottom left). As expected, thymocytes from uninduced-*K18* mice stained with both CD4-PE and CD8-FITC showed normal distribution of CD4+, CD8+, and CD4+CD8+ T cell populations (Fig 4C, top right), and WT CD4+ thymocytes did not express GFP/YFP (Fig 4C, top left). Finally, we analyzed thymocytes from *R26YFP;β-actin Cre* mice and demonstrated the presence of GFP/YFP+ thymocytes (Fig 4C, bottom right). This suggests that transgene was not expressed in thymocytes of *K18* mice.

### Inactivation of Rb-TS in K18+ TEC promotes lymphoid proliferation non-cell-autonomously

**Fig 5:**
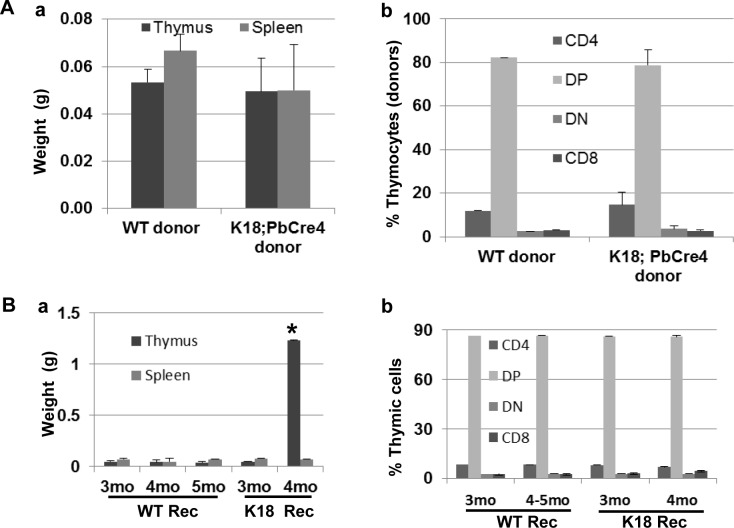
Rb-TS inactivation in K18+ TEC extrinsically regulates thymic hyperplasia. (A) Bone marrow cells including hematopoietic stem and progenitor cells and mature hematopoietic cells (erythroid, myeloid, B cells), as well as very few stromal cells of mesenchymal and endothelial lineages were obtained from Ly5.1+ wildtype (*WT*, n = 7) and *K18;PbCre4* (n = 9) mice, and—transplanted into 3-month old Ly5.2+ lethally irradiated *WT* recipients. There is no statistical difference between *WT* and *K18;PbCre4* donor group. (a) Weight of thymus and spleen in the reconstituted recipients with either *WT* or *K18;PbCre4* bone marrow cells as donor. (b) Percentage of thymocytes derived from *WT* or *K18;PbCre4* donors. DP: double positive (CD4+CD8+), DN: double negative (CD4-CD8-). Data is presented as mean ± SEM. (B) Bone marrow cells obtained from Ly5.2+ *WT* mice were transplanted into 3-month old Ly5.1+ irradiated *K18;PbCre4* recipients (*K18* Rec, n = 5; three recipients were found dead without thymus enlargement at 2–3 weeks post transplantation) or *WT* (*WT* Rec, n = 11) recipients. (a) Weight of thymus and spleen of the reconstituted recipients in grams (g), and mo: months post transplantation. * p < 0.001 (4mo *K18* Rec vs. *WT* Rec). (b) Percentage of T cell subpopulations in *WT* Rec and *K18* Rec transplanted with *WT* bone marrow cells by FACS analysis. DP: double positive (CD4+CD8+); DN: double negative (CD4-CD8-).

To demonstrate the hyperproliferation of thymocytes was induced in a non-cell-autonomous manner, we transplanted Ly5.1+ *K18;PbCre4* or *C57BL/6* (*WT*) bone marrow cells into lethally irradiated Ly5.2+ *WT* recipients. There was no difference in weight or cellularity in recipient thymuses at 4–5 month post transplantation (Fig 5Aa and S4A Fig). CD4 and CD8 profiles were also similar between chimeras with *K18;PbCre4* and *WT* donors (Fig 5Ab). Reciprocal transplants of Ly5.2+ *WT* bone marrow cells into 3-month old Ly5.1+ irradiated *K18;PbCre4* recipients showed that the thymuses of reconstituted *K18* recipients were significantly enlarged at 4-month post transplantation compared to *WT* recipients (Fig 5Ba), indicating that the hyperplasia of lymphoid cells in induced- and lethally irradiated-*K18* recipients was due to a non-cell-autonomous effect (extrinsic) of TEC. The CD4/CD8 profile was not altered in *K18* recipients (Fig 5Bb) compared to that in *WT* recipients, suggesting that Rb-TS inactivation in K18+ TEC affected the TEC proliferation without disrupting the TEC differentiation and T cell development. This is unlike the effect of Pten deletion in TEC that loss of Pten in TEC resulted in a smaller thymus and T cell development was disturbed (increased CD8+ T cells) [30]. Interestingly, we found significant decrease of splenic and bone marrow B220+ cells in *K18* recipients compared to *WT* recipients (S4B Fig), which might explain why spleen weight was not changed although splenic CD4+ and CD8+ cells were increased in induced-*K18* mice. The mechanism by which splenic and bone marrow B220+ cells were decreased is unknown.

Garfin et al. showed that although RB and its family members were deleted in both lymphoid lineage and thymic stroma using Mx1-Cre mice, the enlarged thymus phenotype was due to the proliferation of thymic stroma [23]. However, it was not known what cell subtype in the stroma caused the non-cell-autonomous proliferation of the thymocytes. Our data indicate that inactivation of Rb-TS in K18+ subpopulation of thymic epithelial cells is sufficient to drive the proliferation of both cTEC and mTEC cell-autonomously and T lymphocytes non-cell-autonomously. However, we cannot exclude the possibility that other subpopulations of TEC might play a similar role in thymus proliferation. For example, K19 is predominantly expressed in medullary thymic epithelial cells (S1A Fig), and we observed a similar thymic hyperplasia in a K19-driven T_121_ mouse model [24]. The medium survival of K19-T_121_ mice for thymic hyperplasia-specific cause of death was longer than K18-driven T_121_ mice (7 month vs. 3 month), which was likely due to fewer K19+ T_121_-expressing cells. Furthermore, overexpression of cell cycle regulators (e.g. cyclin D1 or D2) in K5+ mTEC also led to the similar phenotype including cTEC, mTEC and T cell proliferation [29, 31–33]. Thus, disrupting cell cycle regulators (e.g. Rb or cyclin D1) in either K18+, K5+, or K19+ TEC can result in thymic hyperplasia. This suggests that different subpopulations of thymic epithelial cells may have overlapping functions on regulation of T cell proliferation and maturation and control of thymus size.

The precise mechanism governing epithelial control on lymphoid proliferation in this model is unknown. Studies have shown a critical role of transcription factors on the regulation of TEC development (e.g. Foxn1, Tbx1, Pax1, p63, and Cbx4) [34–39]. As a central regulator of the thymic stroma, it has been shown that Foxn1 is negatively regulated by Rb, and Foxn1 expression is required for the thymic expansion in Rb family mutant mice [23], indicating the importance of Rb family proteins in thymic proliferation. Moreover, IL7 and Notch signaling produced by TEC have been demonstrated as key regulators of thymocytes proliferation. It has been shown that IL7 induces thymocyte proliferation in a dose-dependent manner *in vitro* [40], and activation of Notch signaling by its ligands DL1 or DL4 potentiates IL-7- induced proliferation and survival of T cell precursors [41]. In addition, genetic deletion of DL-like 4 (DLL4) or Notch 1 in TEC or pharmacological inhibition of Notch signaling pathway by DLL4-specific antibody in mice led to a dramatic decrease in thymic size and cellularity [42–45]. This demonstrates the importance of TEC on the regulation of thymocyte proliferation. Recently, IL7-producing cells have been identified in a reporter mouse [46]. The fact that both cortical and medullary thymic epithelial cells can produce IL7 indicates a pivotal role of IL7 on various stages of T cell development. Thus, non-cell-autonomous regulation of thymocytes proliferation and development by thymic epithelial cells is evident. Here our study suggests that Rb inactivation in K18+ thymic epithelial cells affects thymocytes proliferation but not maturation.

## Conclusions

Our data suggest that Rb functions to regulate the proliferation of thymic epithelial cells, and inactivation of Rb and its family proteins in K18+ TEC extrinsically promotes the hyperproliferation of thymocytes, without affecting T cell development. Future studies will be aimed to further examine whether the modified K18+ TEC are functioning to produce molecules of known functional importance (e.g. AIRE, DLL4, IL-7, or kit ligand) and to promote correct positive and negative selection or tolerance, and whether T cells produced in contact with these modified epithelial cells are functionally normal. It is also our great interest to assess whether genetically modified K18+ thymic epithelial cells can be used to replace OP9-DL1 or OP9-DL4 co-culture system for supporting T cell development *in vitro* [47–52].

## Supporting information

S1 Fig Representative images of immunostaining in *WT* or induced-*K18* thymuses. (A) IHC staining of K5 and K19 antigens in *WT* thymic cortex and medulla. (B) T_121_ IHC staining in thymic cortex and medulla in *K18;β-actin Cre*, *K18;R26CreER*, and *K18;PbCre4* mice. (C) T_121_ IHC staining in thymuses of 1 and 2 month old *K18;PbCre4* mice. Left panel: without primary antibody control. mo: month.
(TIF)

S2 Fig Characterization of thymuses with Rb-TS inactivation in K18+ TEC. (A) Cre mRNA levels in *PbCre4* males by RT-PCR. Total RNA was extracted from thymus, bladder, and prostate (Pr, as positive control). HPRT was used as loading control. (B) Representative flow cytometry plots of CD4 and CD8 expression in thymocytes from uninduced-*K18* and induced-*K18* mice. Only 2% of induced-*K18* thymuses showed altered CD4 and CD8 profile. (C) Representative immunofluorescence images of T_121_ (green), K18 (yellow), K5 (red), and DAPI (blue) in cortex (C) and medulla (M) delineated by white dotted lines in induced-*K18;Cre* thymus. Right: higher magnification of left image. (D) Representative H.E. image of lymphoma developed in 2% of induced-*K18* mice. (E) Representative images of T_121_ (green) and K18 (red) immunostaining in cultured thymic stroma cells derived from *K18;PbCre4* mice demonstrating that T_121_ was expressed in K18 positive cells. Nucleus was counter-stained with DAPI as blue.
(TIF)

S3 Fig Immunofluorescence staining of induced-*K18;Cre* thymus. Representative immunofluorescence images of Ki67 (green), K18 (red), and DAPI (blue) in thymic cortex and medulla. *Cells are positive for both Ki67 and K18.
(TIF)

S4 Fig Bone marrow transplantation study. (A) Bone marrow cells from Ly5.1+ *K18;PbCre4* and *C57BL/6* wildtype (*WT*) donors were transplanted into 3 month old Ly5.2+ lethally irradiated *WT* recipients. Total thymic cellularity in reconstituted recipients with either *WT* or *K18;PbCre4* bone marrow cells as donor compared to intact *WT* control. (B) Bone marrow cells from Ly5.2+ C57BL/6 *WT* donors were transplanted into 3 month old Ly5.1+ lethally irradiated *K18;PbCre4* (*K18*) and *WT* recipients (Rec). Flow cytometry analysis showed percentage of B220+ cells in spleen (top) and bone marrow (BM, bottom) of *WT* Rec and *K18* Rec. *WT* Rec were 4–5 month post transplantation, and K18 Rec were 4 month post transplantation. mo: months post transplantation. P < 0.05 is considered statistically significant.
(TIF)

S1 Text Supplementary methods including animals, RT-PCR and CBC analysis. (DOCX)
