# Supplementary figures and images for: RB inactivation in keratin 18 positive thymic epithelial cells promotes non-cell autonomous T cell hyperproliferation in genetically engineered mice
Source: PLoS One. 2017 Feb 3;12(2):e0171510. doi: 10.1371/journal.pone.0171510 (PMC5291521; doi:10.1371/journal.pone.0171510)

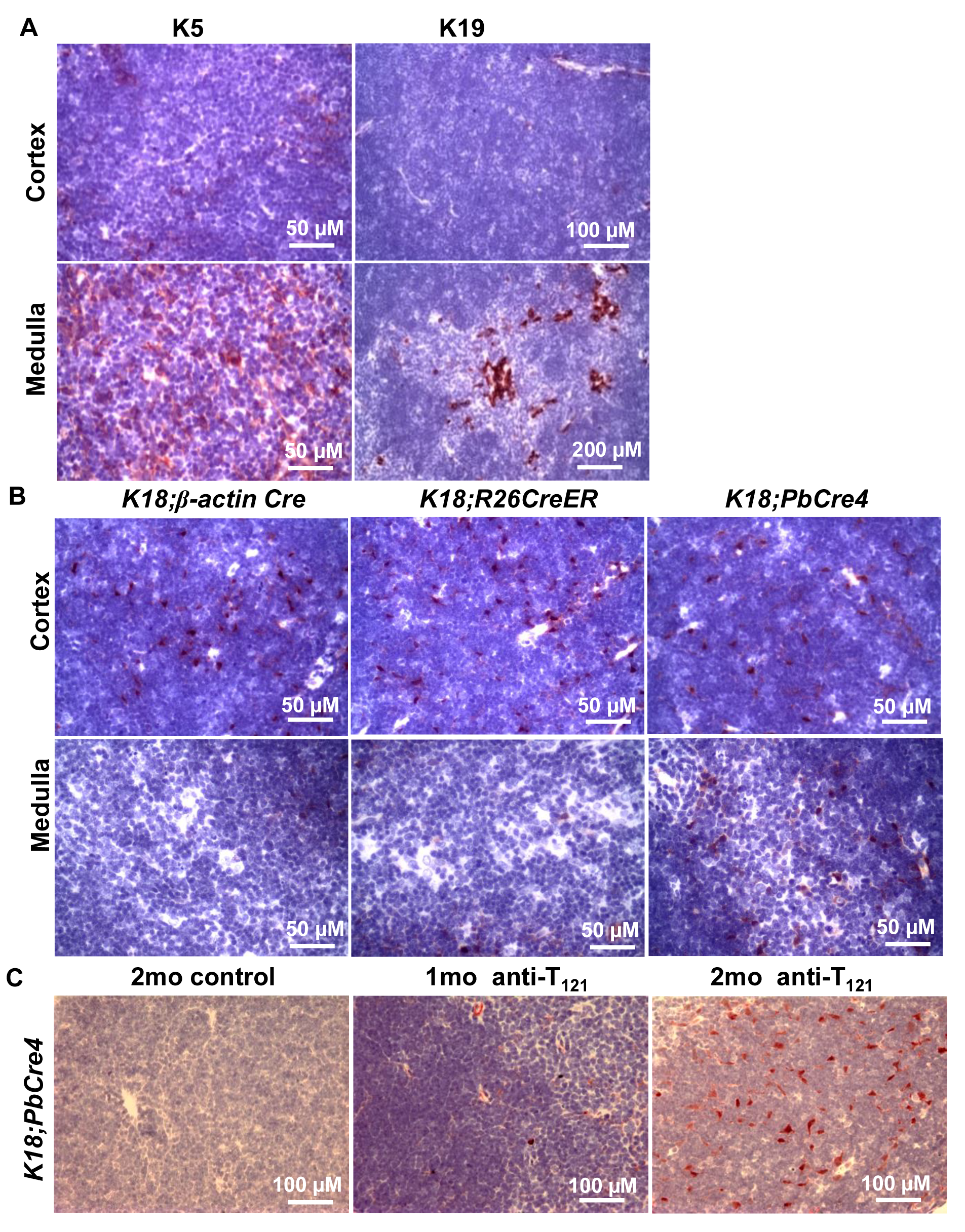

Supplement: S1 Fig — (A) IHC staining of K5 and K19 antigens in WT thymic cortex and medulla. (B) T121 IHC staining in thymic cortex and medulla in K18;β-actin Cre, K18;R26CreER, and K18;PbCre4 mice. (C) T121 IHC staining in thymuses of 1 and 2 month old K18;PbCre4 mice. Left panel: without primary antibody control. mo: month. (TIF) [file pone.0171510.s001.tif]

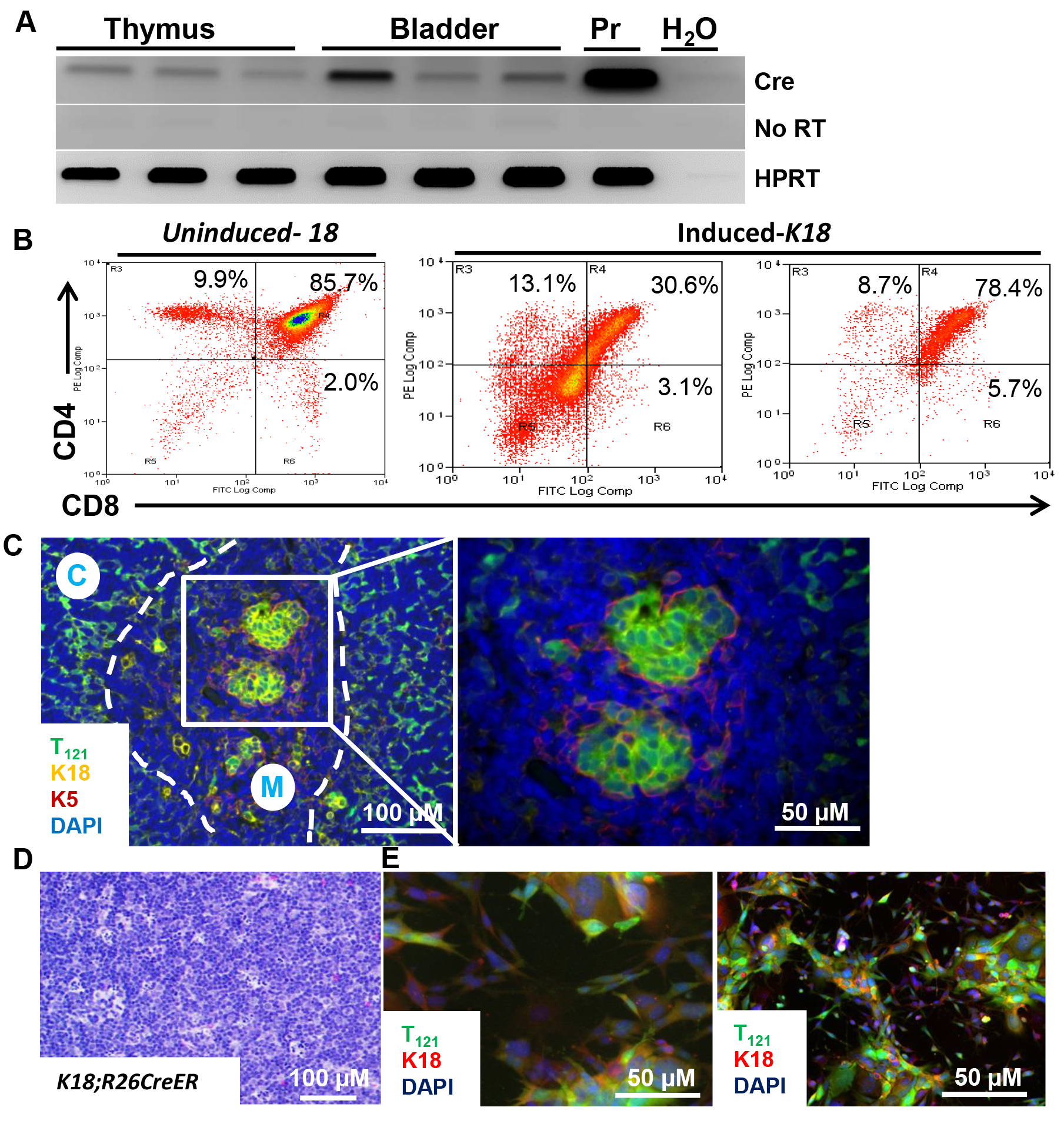

Supplement: S2 Fig — (A) Cre mRNA levels in PbCre4 males by RT-PCR. Total RNA was extracted from thymus, bladder, and prostate (Pr, as positive control). HPRT was used as loading control. (B) Representative flow cytometry plots of CD4 and CD8 expression in thymocytes from uninduced-K18 and induced-K18 mice. Only 2% of induced-K18 thymuses showed altered CD4 and CD8 profile. (C) Representative immunofluorescence images of T121 (green), K18 (yellow), K5 (red), and DAPI (blue) in cortex (C) and medulla (M) delineated by white dotted lines in induced-K18;Cre thymus. Right: higher magnification of left image. (D) Representative H.E. image of lymphoma developed in 2% of induced-K18 mice. (E) Representative images of T121 (green) and K18 (red) immunostaining in cultured thymic stroma cells derived from K18;PbCre4 mice demonstrating that T121 was expressed in K18 positive cells. Nucleus was counter-stained with DAPI as blue. (TIF) [file pone.0171510.s002.tif]

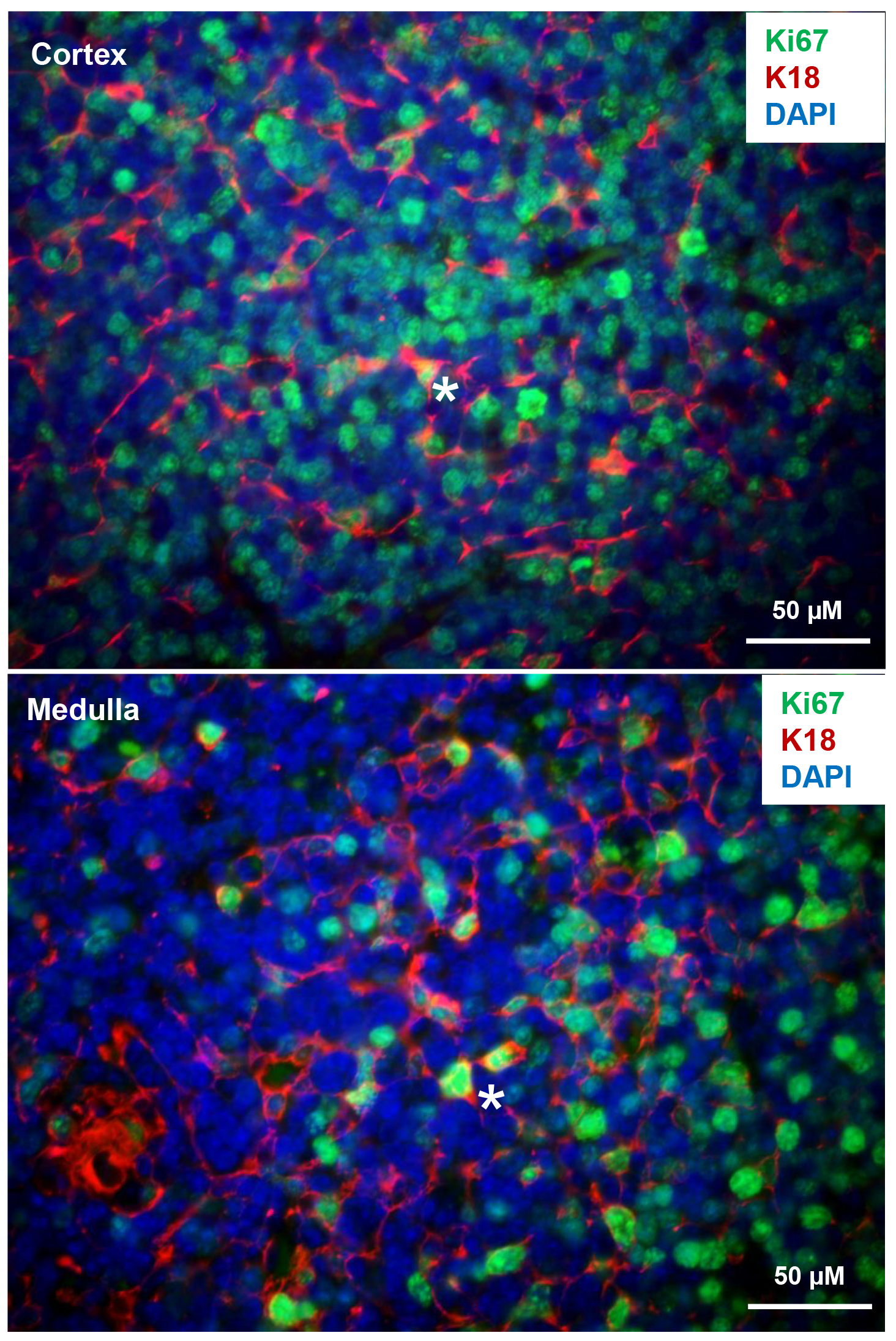

Supplement: S3 Fig — Representative immunofluorescence images of Ki67 (green), K18 (red), and DAPI (blue) in thymic cortex and medulla. *Cells are positive for both Ki67 and K18. (TIF) [file pone.0171510.s003.tif]

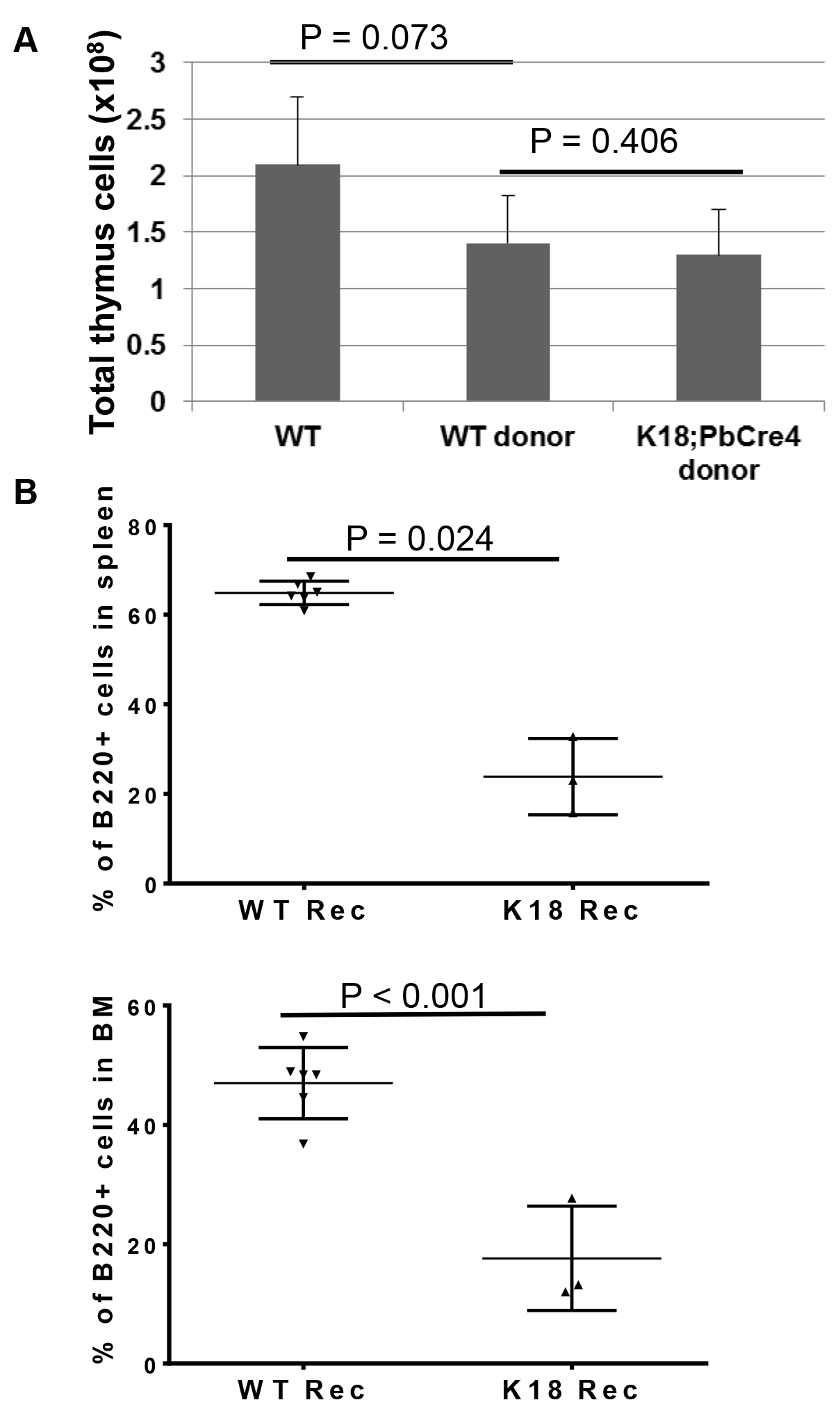

Supplement: S4 Fig — (A) Bone marrow cells from Ly5.1+ K18;PbCre4 and C57BL/6 wildtype (WT) donors were transplanted into 3 month old Ly5.2+ lethally irradiated WT recipients. Total thymic cellularity in reconstituted recipients with either WT or K18;PbCre4 bone marrow cells as donor compared to intact WT control. (B) Bone marrow cells from Ly5.2+ C57BL/6 WT donors were transplanted into 3 month old Ly5.1+ lethally irradiated K18;PbCre4 (K18) and WT recipients (Rec). Flow cytometry analysis showed percentage of B220+ cells in spleen (top) and bone marrow (BM, bottom) of WT Rec and K18 Rec. WT Rec were 4–5 month post transplantation, and K18 Rec were 4 month post transplantation. mo: months post transplantation. P < 0.05 is considered statistically significant. (TIF) [file pone.0171510.s004.tif]
