# Supplementary material for: RB inactivation in keratin 18 positive thymic epithelial cells promotes non-cell autonomous T cell hyperproliferation in genetically engineered mice
Source: PLoS One. 2017 Feb 3;12(2):e0171510. doi: 10.1371/journal.pone.0171510 (PMC5291521; doi:10.1371/journal.pone.0171510)
Supplement: S1 Text — (DOCX) [file pone.0171510.s005.docx]

**RB Inactivation in Keratin 18 Positive Thymic Epithelial Cells Promotes Non-Cell Autonomous T Cell Hyperproliferation in Genetically Engineered Mice**

Yurong Song^1^, Teresa Sullivan^1^, Kimberly Klarmann^1,2^, Debra Gilbert^1^, T. Norene O’Sullivan^1^, Lucy Lu^1^, Sophie Wang^1^, Diana C. Haines^3^, Terry Van Dyke^1^, and Jonathan R. Keller^1,2 *^

**Supplementary Methods**

**Animals**

K18 is widely expressed in epithelial cells. Thus, inactivation of Rb-TS in K18 cells by *β-actin Cre* or *R26CreER* led to hyperplasia not only in thymus but also in other epithelial tissues (e.g. mammary gland, ovarian surface epithelium, intestine, and prostate) [1]. However, because these mice developed severe thymic epithelial hyperplasia and subsequent lymphoid hyperplasia, they all died from life-threatening **thoracic pressure** of enlarged thymuses.

**RT-PCR**

Tissues or cells were collected and minced, then suspended in 1.5ml Eppendorf tubes containing 1ml of Tri-Reagent (Molecular Research Center, Inc., Cincinnati, OH). Bone marrow cells (BMC) were flushed from femurs using 27 gauge needles and DMEM with 5% FBS. BMC were pelleted by centrifugation, and then suspended in Tri-Reagent. Total RNA was extracted following manufacture’s instruction, and reverse transcribed into cDNA. PCR was performed as described previously [2].

**CBC analysis**

Complete hematology profile in heparinized whole blood of wildtype and K18;Cre mice was analyzed using the CBC Hemavet blood counter calibrated for mouse by Pathology/ Histotechnology Laboratory at Leidos Biomedical Research, Inc..

**References**

1. Song Y, Gilbert D, O'Sullivan TN, Yang C, Pan W, et al. (2013) Carcinoma initiation via RB tumor suppressor inactivation: a versatile approach to epithelial subtype-dependent cancer initiation in diverse tissues. PLoS One 8: e80459.

2. Hill R, Song Y, Cardiff RD, Van Dyke T (2005) Heterogeneous tumor evolution initiated by loss of pRb function in a preclinical prostate cancer model. Cancer Res 65: 10243-10254.
